# Current News and Opinion

**Published:** 1887-10

**Authors:** 


### Current News and Opinion.

### "THE WORLD MOVES."

In the course of a brief conversation with President Davis, of the International Medical Congress, during its recent session in Washington, after congratulations and expressions of satisfaction at the success of the meetings, reference was made to the apathy or indifference manifested by certain prominent physicians in New York and Philadelphia, who not only refused to give their aid and presence to further the interests of the Congress, but in some instances used their influence to defeat its objects, in hopes to make it a failure. "Well, the world still moves" remarked the worthy President, "and their loss is greater than ours." Without their aid, and despite their wet blanket treatment, the Congress made its mark of success as every person who attended will testify, and all who have read reports of its deliberations must be convinced.

In regard to the controversies and factional wrangles for control of the Ninth Congress during the period of its inception, we have here no criticism to make. So long as the fact was settled that the Congress would meet in Washington at a fixed time, all sectional interests and party feelings should have been held in restraint, and all parties should have used their best efforts to make the meeting of the Congress worthy of our country, and thus show to the world the great strength and excellent standing of the Medical Profession of America. Such occasions seldom come to us, and surely the outcroppings of jealousy in the contests of rivalry should not have been so sorely manifested. "Rule or ruin," is a poor motto. It is a sacrafice of everything for the glory of one's self; a disregard of the feelings of others and the general good of mankind. In its results it is like the policy of excising the nasal protuberance in order to spite the countenance. "More's the pity!" C. E. F.

### THE ORIGIN OF EROSIONS ON THE CROWNS OF TEETH.

Prof. Busch, as a result of his experience, believes that defects in the enamel of the teeth, which is commonly known as erosions, are due to certain laws, not only with reference to their localization upon the crown, but also with reference to their occurrence upon particular teeth in the set. These furrows and depressions affect only the superficial portions of the crown, while the part which is near the root may always retain its normal consistency. These erosions never affect a single tooth or several teeth without any regard to position or other conditions, but always affect, in any given case, those which are developed in the jaw at the same period, and hence those groups are most frequently and most decidedly affected whose crowns develop earliest. The first molar stands foremost in the list, its crown being partly developed as early as the time of birth. The incisors and canines come next, their crowns developing during the first few months of life. The bicuspids are seldom eroded, their crowns developing, for the first, at the end of the second year of life, for the second, a year later. The last two molars, the crown of the first of which begins to develop at the end of the fourth year, and the second between the eighth and ninth years, are always free from erosions. The milk-teeth may be of irregular form and bad structure, but they never show the peculiarity of the erosion. Hutchinson's assumption that erosions are attributable to syphilis is considered unwarranted, as otherwise they should appear upon the milk-teeth. That assumption also leaves unexplained the fact that the erosions appear upon particular groups of teeth, as has been already stated. They are also absent in many cases of undoubted hereditary syphilis and present in many individuals in which no trace of hereditary syphilis is discoverable. In hereditary syphilis, rachitis, and scrofula, the milk-teeth are apt to be soft and friable, but the erosions of the permanent teeth cannot be explained as peculiarities of those diseases. The author believes that they are the result of disease which affects the individual at the time that the crowns are beginning to develop. Among the diseases which may have such an influence, eclampsia, meningitis and severe attacks of choking, in consequence of whooping cough (for example), are mentioned. A single severe attack of convulsions in the first year of life might be sufficient to produce erosions on the teeth, the crowns of which were developing at that time. The theory is that the disease would cause a temporary suspension of the growth of the tooth, that it would soon begin again to grow vigorously, but that the interval would be marked by a furrow or erosion. After the first year of life cerebral disorders are much less frequent, and hence the infrequency of erosions upon teeth which are developed after that time. As erosions are believed to be so frequently caused by convulsive conditions, the author suggests the propriety of speaking of *teeth convulsions.* From one to two per cent. of all human beings have erosions on the teeth. A tendency to caries is manifest when they are present.

—*Archives of Pediatrics.*

---

### SOUTHERN DENTAL ASSOCIATION.

The following named members were elected as officers for the coming year:

*President*—B. H. Catching, Atlanta, Ga.

*First Vice-President*—J. H. Prewitt, Madisonville, Ky.

*Second Vice-President*—W. N. Morrison, St. Louis, Mo.

*Third Vice-President*—J. Hall Moore, Richmond, Va.

*Corresponding Secretary*—J. Y. Crawford, Nashville, Tenn.

*Recording Secretary*—L. P. Dotterer, Charleston, S. C.

*Treasurer*—H. S. Lowrance, Athens, Ga.

*Executive Committee*—Drs. Edwards and Doyle, Louisville, Ky., W. Dancy, Jacksonville, Fla.

Place of meeting, Louisville, Ky., in joint session with American Dental Association.

Time of meeting, fourth Tuesday in August. All details left to Joint Committee.

---

### OHIO STATE DENTAL SOCIETY.

The next annual meeting of the Ohio State Dental Society will be held in Springfield, on Wednesday, October 26th, and continue three days.

J. R. Callahan, Secretary.

### UNION DENTAL CONVENTION.

The nineteenth annual dental convention of the Seventh and Eighth District Dental Societies will occur on Tuesday, October 25th, in Buffalo.

This year the Sixth District Dental Society will unite in the convention, and assurances have been received that a large delegation from the Fifth District Society will be in attendance also.

The sessions of the convention will be held in the new Library Building, and the committee express with great confidence the conviction that the occasion will be more profitable and interesting than usual. Some new features are contemplated, but complete arrangements are not yet perfected.

The circular giving full particulars will soon be issued. Let all attend who can.

---

### FIFTH DISTRICT DENTAL SOCIETY.

The Fifth District Dental Society of the State of New York will hold its nineteenth semi-annual meeting at Syracuse, Tuesday and Wednesday, October 11 and 12, 1887. The session will be called to order at 2 P. M. at the Vanderbilt House. Applications for membership in the society should be made on or before the day of meeting to the Chairman of the Board of Censors, or to the Recording Secretary. The Board of Censors will be in attendance to examine candidates for admission to the society. Members of the profession from other societies are cordially invited to be present and take part in the discussions.

C. J. PETERS, Rec. Sec.

---

### NEW ENGLAND DENTAL SOCIETY.

The next meeting of the New England Dental Society will be held in Boston, on Wednesday, Thursday, and Friday, the 5th, 6th and 7th of October. As this is its twenty-fifth anniversary, the society proposes to celebrate its silver wedding by a large and interesting gathering. A. M. DUDLEY, Sec'y.

---

### CONNECTICUT VALLEY DENTAL SOCIETY.

The Connecticut Valley Dental Society will hold its next annual meeting at Hotel Warwick, Springfield, Mass., October 27th and 28th. A cordial invitation is extended to all dentists. GEO. A. MAXFIELD, D. D. S., Sec'y.

---

### ORIENT !

An opportunity is offered to secure the dental practice at a foreign court. The applicant must be an American dentist, first-class in every respect.

Particulars by addressing P. L. FOOTE, D. D. S.,
Poughkeepsie, N. Y.

---

DR. H. W. PARSONS, of Wamego, Kansas, exhibited at the Congress a number of ingenious contrivances, among which were an air pump to be attached to the dental engine, and which worked a saliva ejector, a hot air injector and a chip blower. If the hot air injector is as practical as it appeared to be, it will supply a decided want, for the present tendency is toward the use of hot air as the most effectual obtundent for sensitive dentine.

The National Association of Dental Faculties met at the Ebbitt House, in Washington, on Saturday, Sept. 3d. The Association has accomplished good in the past, but there is much for it yet to do if it proposes to raise all the colleges represented to the standard recommended by the leading societies. In the past there has seemed to be a disposition to whitewash too freely. Leading schools have violated the code of agreement and departed from the established regulations with impunity. If the Association is to retain the confidence of the profession, it must hew to the line.

The officers elected for the ensuing year were:

*President*—A. O. Hunt, Iowa City.

*Vice-President*—Thos. Fillebrown, Portland, Me.

*Secretary*—J. E. Cravens, Indianapolis, Ind.

*Treasurer*—A. W. Harlan, Chicago, Ill.

*Executive Committee*—Frank Abbott, New York City; J. Taft, Cincinnati, O.; S. H. Guilford, Philadelphia, Pa.

---

We are pleased to announce the election of our old (not in years of age, but in length of friendship) friend, Dr. B. H. Catching, of Atlanta, Ga., editor of *The Southern Dental Journal*, to the presidential chair for the ensuing year. Especially is this gratifying on account of the many efforts of Dr. Catching in behalf of the Southern Dental Association, and also from the fact that a vain effort was made to elect to that position a gentleman who obtained his degree of D. D. S. from the so-called Delaware (University) College.—*American Journal Dental Science.*

This demands an explanation. Does the Southern Dental Association admit to membership dentists who have been caught holding boughten diplomas? Will it accept "graduates" of that filthy Delavan (Delaware?) fraud? We do not know who the gentleman referred to may be, but some one should rise and explain. The "Southern" is too representative an association, and is made up generally of men of too high professional character to rest under this stigma.

---

Apropos to the present Congress it may be interesting to note that the first International Medical Congress was held in Paris, in 1867; the second in Florence, in 1869; the third in Vienna, in 1873; the fourth in Brussels, in 1875; the fifth in Geneva, in 1877; the sixth in Amsterdam, in 1879; the seventh in London, in 1881; and the eighth in Copenhagen, in 1884. It was originally intended that the Congress should be held every second year, but at the meeting in London it was decided to hold the meeting once in three years. The Congress met this week for the first time on American soil. It will probably be many years before it will again cross to this side of the Atlantic.

Professor N. S. Shaler, whose articles on Earthquakes, Cyclones, and Forests in *Scribner's Magazine* have attracted wide attention, contributes to the October number of that periodical a similar paper on "Caverns and Cavern Life" which is richly illustrated.

---

There was no paper read before the Medical Congress which produced a more profound impression than the address of Prof. Mariano Semmola, of Naples, Italy, upon "Bacteriology." The paper will be found in full in the *New York Medical Journal.*

Prof. Busch, of the University of Berlin, made a most astonishing exhibition of pathological changes in the tusks of elephants. Such a collection, we are certain, was never made before. There were abscess cavities in the dentine, pulp nodules half-a-foot long, and many cases of so-called secondary dentine. He showed that he knew what he was talking about when he asserted that the points of the tusks in young elephants are covered with a plate of enamel. This was disputed by Dr. Atkinson, who intimated that the professor meant eels when he said elephants, but who had nothing to say in response when Prof. Busch produced an indisputable specimen.

---

Dr. William C. Wile, who removed from Newtown, Conn , to Philadelphia to occupy the chair of Electro-Theraputics and Nervous Diseases in the Medico-Chirurgical College of Philadelphia, and who became one of its editors upon the establishment of *The Medical Register*, has dissolved those connections and formed a new one—a matrimonial one—and will give up all for domesticity. He will settle near the former home of his bride, in Danbury, Conn. That wife must be a charming one, to overcome the wishes of a whole profession. Dr. Shoemaker assumes sole charge of *The Register*, and he is quite equal to the task.

---

Dr. N. A. Randolph, the senior editor of the Philadelphia *Medical and Surgical Reporter*, was drowned at Atlantic City in the endeavor to save the life of another on August 21st Dr. Randolph had undertaken a very difficult task, that of succeeding the brilliant Dr. D. G. Brinton as senior editor of the *Reporter*, and had, in connection with Dr. Dulles, scored a decided success. His death is greatly regretted.

---

Frank W. Sage, D. D. S., has for two years been the dental editor of *The Cincinnati Medical and Dental Journal*, and he has always filled his department with fresh and readable matter. In the last number we notice with regret the announcement that he has severed his connection with that journal, and that hereafter it will be under the charge of another.

---

Dr. Geo. H. Cushing has accepted the chair of Principles and Practice of Dentistry in the Chicago Dental College, and will enter upon the discharge of his duties at once. His long experience as a practitioner and his deep interest in educational matters admirably qualify him for the work of a teacher.

---

The next meeting of the American Dental Association will be held in connection with the Southern Dental Association, at Louisville, Ky., on the fourth Tuesday in August, 1888. This should make the largest and most influential gathering of dentists which America has yet seen.

---

The Medical Register of Philadelphia issued a daily edition during the meeting of the International Congress, and the series gives a very accurate *resumé* of the proceedings. They will be valuable for future reference.

---

The Tenth International Medical Congress will meet in Berlin in 1890.

